# Colonic Oxidative and Mitochondrial Function in Parkinson's Disease and Idiopathic REM Sleep Behavior Disorder

**DOI:** 10.1155/2017/9816095

**Published:** 2017-06-04

**Authors:** C. Morén, Í. González-Casacuberta, J. Navarro-Otano, D. Juárez-Flores, D. Vilas, G. Garrabou, J. C. Milisenda, C. Pont-Sunyer, M. Catalán-García, M. Guitart-Mampel, E. Tobías, F. Cardellach, F. Valldeoriola, A. Iranzo, E. Tolosa

**Affiliations:** ^1^Muscle Research and Mitochondrial Function Laboratory, Cellex-IDIBAPS, Faculty of Medicine and Health Sciences, University of Barcelona, Internal Medicine Department, Hospital Clínic of Barcelona, CIBERER U722, Barcelona, Spain; ^2^Neurology Service, Hospital Clínic of Barcelona, IDIBAPS, CIBERNED, Barcelona, Spain; ^3^Movement Disorder Unit, Neurology Service, Hospital Clínic of Barcelona, IDIBAPS, CIBERNED, Barcelona, Spain

## Abstract

**Objective:**

To determine potential mitochondrial and oxidative alterations in colon biopsies from idiopathic REM sleep behavior disorder (iRBD) and Parkinson's disease (PD) subjects.

**Methods:**

Colonic biopsies from 7 iRBD subjects, 9 subjects with clinically diagnosed PD, and 9 healthy controls were homogenized in 5% w/v mannitol. Citrate synthase (CS) and complex I (CI) were analyzed spectrophotometrically. Oxidative damage was assessed either by lipid peroxidation, through malondialdehyde and hydroxyalkenal content by spectrophotometry, or through antioxidant enzyme levels of superoxide dismutase-2 (SOD2), glutathione peroxidase-1 (Gpx1), and catalase (CAT) by western blot. The presence of mitochondrial DNA (mtDNA) deletions was assessed by long PCR and electrophoresis.

**Results:**

Nonsignificant trends to CI decrease in both iRBD (45.69 ± 18.15; 23% decrease) and PD patients (37.57 ± 12.41; 37% decrease) were found compared to controls (59.51 ± 12.52, *p*: NS). Lipid peroxidation was maintained among groups (iRBD: 27.46 ± 3.04, PD: 37.2 ± 3.92, and controls: 31.71 ± 3.94; *p*: NS). Antioxidant enzymes SOD2 (iRBD: 2.30 ± 0.92, PD: 1.48 ± 0.39, and controls: 1.09 ± 0.318) and Gpx1 (iRBD 0.29 ± 0.12, PD: 0.56 ± 0.33, and controls: 0.38 ± 0.16) did not show significant differences between groups. CAT was only detected in 2 controls and 1 iRBD subject. One iRBD patient presented a single mtDNA deletion.

## 1. Introduction

The main pathological features of Parkinson's disease (PD) are the loss of nigral dopaminergic neurons of the central nervous system (CNS) and the presence of aggregates of abnormal *α*-synuclein, so-called Lewy bodies and Lewy neurites, in remaining neurons. Lewy type pathology also emerges in the peripheral autonomous nervous system (PANS) [1, 2] and is known to occur in early, prodromal stages. Abnormal aggregates of synuclein are not uncommon in the enteric neurons of PD patients and the evidence of a prion-like behavior of synuclein [3] has focused the attention on the assessment of the enteric nervous system in PD in line with the hypothesis that the gut is the initial site of synuclein aggregation [4–6]. Since abnormal aggregates of synuclein are found in the enteric neurons in PD, a colonic biopsy could provide access to a unique tissue to study pathophysiological aspects of the disease [4–6].

Dysfunctions in mitochondrial metabolism, respiration, bioenergetics, and dynamics are suggested to play a significant role in the development of both idiopathic and genetic forms of PD [7–9]. Mitochondrial alterations related to this condition have been found in the CNS and also described in other tissues and structures such as fibroblasts [10], platelets [11], and skeletal muscle [12, 13].

Complex I (CI) dysfunction was the first depicted association between mitochondria and PD [14, 15]. Inhibition of CI can lead to the degeneration of neurons by a number of mechanisms [16]. Although the electron transport chain is a highly efficient system within mitochondria, electrons can leak from the chain, especially from CI [17] of the mitochondrial respiratory chain (MRC), and react with oxygen to form reactive oxygen species (ROS). ROS production leads to oxidative damage and neuronal death in PD [18]. One of the principal mechanisms of ROS-derived cell damage includes the disruption of the cell membrane through lipid peroxidation. Furthermore, *α*-synuclein oligomers can promote lipid peroxidation and a decline of endogenous antioxidants [19]. Failure of the antioxidant enzyme machinery, such as superoxide dismutase (SOD), glutathione (GSH) peroxidase (Gpx), and catalase (CAT), could also be involved in PD, as deficiencies in such antioxidant enzymes have been directly related to this disorder [16]. Lastly, mitochondrial genetic alterations have also been described in PD [20], such as the presence of mitochondrial DNA (mtDNA) deletions, often related to ROS generation.

Although the main molecular features remaining at the core of PD etiopathogenesis are (i) mitochondrial alterations, (ii) oxidative damage, and (iii) the mishandling degradation of proteins [21–23], only unfolded proteins have been assessed at PANS level [4–6] from subjects suffering from idiopathic REM sleep behavior disorder (iRBD), a parasomnia characterized by dream enactment behavior and atonia during REM sleep. iRBD patients are considered to suffer prodromal synucleinopathies since up to 80% of these patients develop a neurodegenerative disease, associated with Lewy body pathology [24–26].

The reported strong association of mitochondrial changes and PD led us to explore some mitochondrial and oxidative parameters in peripheral enteric tissue to potentially contribute to the study of the earliest mechanisms underlying neurodegeneration in PD. Studies on this type may provide evidence of peripheral mitochondrial dysfunction in the earliest stages of PD.

We hypothesized that mitochondrial and oxidative alterations could be present in colonic tissue from subjects with idiopathic iRBD and PD and, thus, in prodromal and clinically manifest PD patients. The aim of the study was to explore peripheral mitochondrial and oxidative function ideally as putative mitochondrial biomarkers for PD.

## 2. Materials and Methods

Patients were recruited from those visited at the Movement Disorders unit and the Sleep Unit of the Hospital Clínic of Barcelona. Demographic and clinical data were collected. Patients with PD and iRBD (the latter confirmed by polysomnography and lack of clinical signs of parkinsonism or cognitive dysfunction) were studied.

Control subjects with no evidence of neurologic disease or dream-enacting behaviors were prospectively and consecutively enrolled in this study. Colonic biopsies by colonoscopy were obtained. Control participants were recruited from the gastroenterology service and indications for colonoscopy included routine preventive cancer screening and follow-up of colonic adenomas, as well as workup for diarrhea. The protocol was approved by the hospital ethical committee on human experimentation before the study initiation and written informed consent for research was obtained from all individuals participating in the study.

Nonmotor symptoms regarding gastrointestinal system were evaluated using the Non-Motor Symptoms Questionnaire (NMSQuest) items 19 to 21. Age at biopsy and disease duration (motor onset in PD patients and abnormal dream-enacting suggesting of iRBD in iRBD patients) were also documented.

Neurological evaluation of participants was carried out by means of the Movement Disorder Society-Unified Parkinson's Disease Rating Scale (MDS-UPDRS) parts I to III, the Montreal Cognitive Assessment to rule out cognitive impairment in iRBD and control subjects.

Colonic biopsies were performed in patients according to the standard procedure of the Gastroenterology Department of Hospital Clínic of Barcelona.

Standard colonoscopies and 3 to 5 biopsies were obtained from ascending, transverse, descending, and sigmoid colon using the standard biopsy forceps, as previously described [27]. Ascending and descending colonic explants were used for culture and sigmoid fragment was cryopreserved at −80°C for further analysis.

### 2.1. Sample Processing

Tissue explants were cut in approximately 4 pieces of 1 mm^2^ and washed 3–5 times with sterilized 1x PBS supplemented with 10% penicillin, streptomycin, and fungizone (PSF). Homogenization of cryopreserved sigmoid colon biopsies was performed using 5% (w/v) mannitol buffer in potter glass at 850 rpm and further centrifuged at 650*g* during 20 minutes. Supernatant was kept and pellet was again resuspended in 5% (w/v) mannitol buffer, homogenized, and centrifuged at the same conditions. Supernatants from both centrifugations were mixed to obtain a mitochondrial-enriched suspension.

### 2.2. Experimental Assays

Total protein content was measured by bicinchoninic acid (BCA) kit assay quantification and run in microplates, by independent measurement of sample dilution duplicates. A set of internal standards were read in parallel together with a reference internal control sample.

For the quantification of the enzymatic assays [citrate synthase (CS) and CI] an internal reference pork muscle homogenate at 2 mg/ml was used. This is the gold standard material to confirm the validation of such spectrophotometric measurements, to better characterize the optimal velocity while maintaining linear kinetics relative to time and enzyme concentration.

Mitochondrial content was measured spectrophotometrically in cuvettes through CS enzymatic activity involved in the Krebs cycle, considered a reliable marker of the mitochondrial mass [28]. This assay was performed by means of the quantification of the product reaction 5,5′ dithiobis 2-nitrobenzoic acid at 412 nm and at 37°C.

The CI enzymatic activity of MRC was measured spectrophotometrically in cuvettes following the quantification of NADH substrate oxidation and using the specific inhibitor rotenone at 340 nm and at 37°C, as reported elsewhere [29].

Either protein content or CS or CI enzymatic assays were performed by standardized procedures following protocols from the national Spanish Network CIBERER (unpublished protocols).

The oxidative damage was determined by the quantification of lipid peroxidation derived products: malondialdehyde and 4-hydroxyalkenals, using the assay kit OxisResearch LPO586 at 586 nm, following manufacturer instructions [30].

SOD2, Gpx1, and CAT antioxidant enzyme protein levels were assessed by western blot as follows. Sample DTT-reduced homogenates were loaded equally using SDS-PAGE (precast 12% gels, Novex; Thermo Fisher Scientific) and transferred by wet transfer onto PVDF membranes (Amersham Hybond, 0.45 *µ*M: GE Healthcare). Membranes were blocked using 5% powdered skim milk-PBS solution for 1 hour (RT) and then incubated with primary antibody in 1 : 1 blocking buffer: PBS 0.4% Tween 20 solution. SOD2 (SIGMA), CAT (AbCam), and Gpx1 (Cell Signalling) were used for immunostaining by 1-hour incubation (RT), followed by 1 : 2000 horseradish peroxidase- (HRP-) conjugated secondary antibodies (Biorad) for 1 hour in 1 : 1 blocking buffer (PBS 0.4% Tween 20 solution). Enhanced chemiluminescence (ECL) substrate (Thermo Fisher Scientific) was used to detect immunoreactive proteins on X-ray film further scanned to quantify band intensity.

MtDNA deletions were determined by long PCR using Long COX-F 5′-TTAGCAGGGAACTACTCCCA-3′ and Long H 5′-CGGATACAGTTCACTTTAGCTACCCCCAAGTG-3′ primers for 10.2 Kb amplicon. After amplification of mitochondrial genome, an electrophoresis was run in a 0.8% agarose gel with red safe.

Statistical analysis was performed using SPSS. Normality of the data was assessed by Kolmogorov-Smirnov test. Nonparametric *U* Mann–Whitney test for independent samples was used. Significance level was set at *p* < 0.05.

## 3. Results

Demographic and clinical data of the participants are summarized in Table 1. A total of 25 adult subjects were enrolled in the study including 9 PD (highest Hoehn-Yahr scale scoring of 2), 7 iRBD, and 9 healthy subjects. iRBD patients were older than PD (*p* = 0.007, Mann–Whitney *U*) whereas the difference was not significant when compared to control subjects (*p* = 0.05, Mann–Whitney *U*). As expected, UPDRS showed a more severe involvement in PD compared to iRBD and control subjects. iRBD and control subjects with a score higher than 0 at motor evaluation (UPDRS III) were known to suffer from nonneurological movement impairment, mainly due to arthrosis. PD patients were more likely to suffer from gastrointestinal symptoms than iRBD (*p* = 0.031, Mann–Whitney *U*).

Cryopreserved homogenates were used for experimental procedures. The presence of neuronal axons was proved in the tissue explants by fluorescence confocal microscopy (Supplementary Material, Figure  S1a, available online at https://doi.org/10.1155/2017/9816095).

Mitochondrial enzymatic activities, oxidative damage, determined by lipid peroxidation, and antioxidant levels and deletions in mitochondrial genome were measured in the cryopreserved sigmoid homogenates.

CS enzymatic activity showed equivalent mitochondrial mass in all the groups (data not shown).

**Figure 1:**
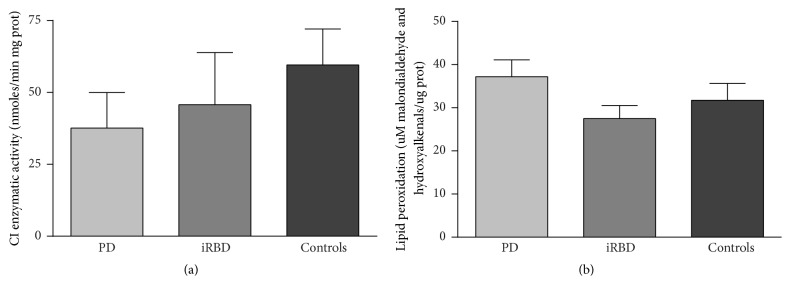
*CI and lipid peroxidation assessment*. (a) CI enzymatic activity of MRC. (b) Oxidative stress analysis through lipid peroxidation measured by malondialdehyde and hydroxyalkenal content. CI, complex I; MRC, mitochondrial respiratory chain; PD, Parkinson's disease; iRBD, idiopathic REM sleep behavior disorder.

No significant differential trends were observed in some of the parameters among groups. Interestingly, mitochondrial CI of MRC was slightly decreased in iRBD and PD patients compared to controls. This trend to decrease was more evident in clinically diagnosed PD patients (Figure 1(a)). Oxidative damage levels measured through lipid peroxidation by malondialdehyde and hydroxyalkenal content were slightly higher in PD but not in iRBD subjects, when compared to the controls (Figure 1(b)). The quantification of the mitochondrial parameters did not render statistically significant differences in any case.

**Figure 2:**
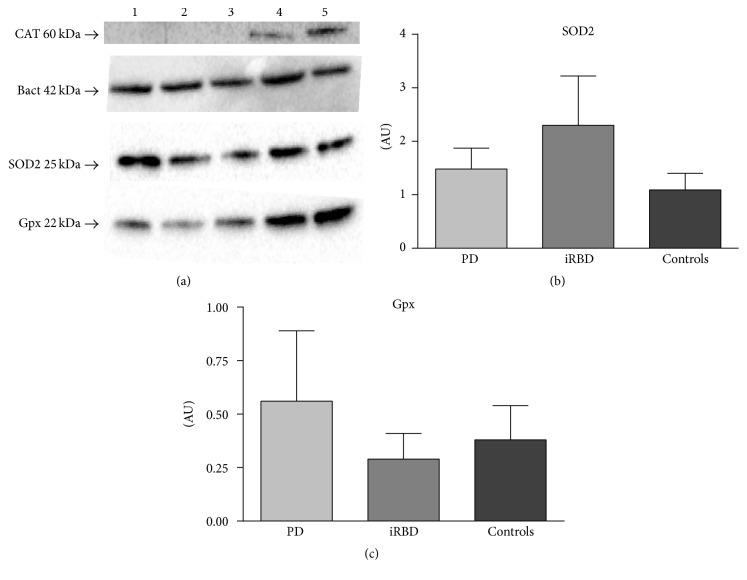
*Antioxidant enzymes quantification* through western analysis of controls (*n* = 9), iRBD (*n* = 7), and PD patients (*n* = 9). (a) Representative blots showing expression levels CAT, SOD2 and Gpx antioxidants, and *β*-actin loading control. Well ID is as follows: (1) control, (2) control, (3) iRBD, (4) iRBD, and (5) control. (b) Quantification of SOD2 levels. (c) Quantification of Gpx levels. AU, arbitrary units; SOD2, superoxide dismutase-2; Gpx, glutathione peroxidase; PD, Parkinson's disease; iRBD, idiopathic REM sleep behavior disorder.

No changes in antioxidant enzyme levels of SOD2 and Gpx1 were found between groups (Figures 2(b) and 2(c), resp.). Antioxidant enzyme CAT was only detectable in 2 controls and 1 iRBD patient. None of the PD patients showed CAT reactive staining.

**Figure 3:**
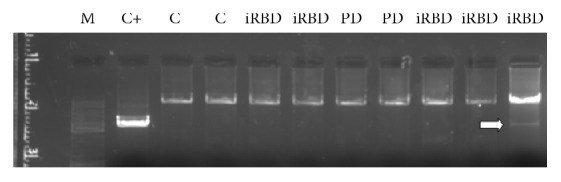
*MtDNA deletions*. 0.8%-agarose gel containing amplified mtDNA. White arrow shows a iRBD patient presenting a unique mtDNA deletion. C, controls; iRBD, idiopathic REM behavior disorder patients; PD, Parkinson's disease patients; C+, internal reference positive control; mtDNA, mitochondrial DNA.

MtDNA deletions were absent, although the presence of a unique mtDNA deletion in a iRBD subject was remarkable, as shown in Figure 3.

## 4. Discussion

Studies on the identification of surrogate in vivo peripheral prognostic markers for PD so far are relevant as they may allow minimally invasive diagnostic procedures and provide a window to early preclinical diagnosis and putative neuroprotective therapies during the prodromal phase of PD [31]. Constipation is very common in the prodromal phase of PD and objective colonic dysfunction has been recently ascertained in PD patients [32]. At a molecular level, the presence of synuclein aggregates in colonic biopsies of PD subjects has been widely discussed as a main diagnostic factor for PD prior to the onset of clinical motor features, although providing controversial results [5, 33], and other differential factors, such as decreased intestinal acetylcholinesterase activity, have been reported in early PD [34]. Although growing attention has been focused on the search for predictive prognostic biomarkers of the disease in the enteric nervous system, no gold standard consensus has been achieved, and mitochondrial and oxidative markers in colonic samples from iRBD and PD patients have not yet been explored.

Despite the wide amount of data on mitochondrial dysfunction associated with PD, our study, reported here, in colonic biopsies of prodromal and clinically diagnosed PD patients did not detect conclusive mitochondrial alterations.

In the colonic tissue of our study subjects mitochondrial mass, estimated by CS enzymatic activity, remained similar in all the groups, suggesting that no relevant changes in mitochondrial biogenesis of the samples occur.

Although CI MRC did not present any significant changes among groups, a trend of decrease of this activity was observable in both prodromal and clinically diagnosed PD groups with respect to controls. These CI enzymatic activity levels were slightly lower in patients with manifest PD. On the other hand, oxidative damage measured by lipid peroxidation tended to not significantly increase in enteric tissue from patients but not in iRBD subjects compared to controls. These tendencies may suggest that decrease of CI enzymatic activity could occur prior to oxidative damage in PD. It is widely known that a MRC dysfunction, especially at CI level, may lead to oxidative damage due to electron leaks in the MRC and the consequent formation of ROS that promotes oxidative stress. In the present study the slight trends in CI enzymatic activity changes are observed in both iRBD and PD patients, suggesting that oxidative damage changes over time could be secondary to CI dysfunction. It is of note that previous studies in PD have pointed out to the presence of oxidative damage in the colonic submucosa either in physiological and pathological conditions [35].

Antioxidant enzyme protein levels did not show significant changes between mitochondrial SOD2 and Gpx1 enzymatic levels between groups and CAT levels were not detectable in most cases, leaving an opened question about whether this was due to an art factual issue or due to a potential relevant molecular meaning relaying on such differential data. Although our findings point out to a lack of an association between endogenous antioxidant changes in PD peripheral tissue, a recent study has described specific plasmatic changes in antioxidant levels and lipid peroxidation as potential promising PD biomarkers [36].

MtDNA deletions have been found in the* substantia nigra* of PD patients or in in vitro models of PD, but they have not been detected in other areas of the brain and it is uncommon to find mtDNA deletions in peripheral tissues. Given the use of colonic biopsies in the present study, it is not surprising that most of the patients did not show any mtDNA deletion and, indeed, the presence of a unique mtDNA deletion of a iRBD subject is remarkable.

In the present study, which included part of the cohort of a previous work [27], only one patient with positively phosphorylated synuclein aggregates in submucosal neurites was included. As discussed by Sprenger et al., the low percentage of positive cases could be related to the site of biopsy (as a rostrocaudal gradient of pathology in the gastrointestinal tract has been suggested by some authors), the possible centripetal distribution of *α*-synuclein pathology (from the peripheral axon to the central neuronal soma), and the depth of the biopsy, which is critical for an appropriate assessment of submucosal autonomic plexus. Unfortunately, due to these possible limitations, any adequate correlation analysis between mitochondrial function and abnormal *α*-synuclein aggregation could be assessed.

In summary, our findings in enteric samples do not demonstrate clear mitochondrial abnormalities in colon biopsies of iRBD or established PD patients. Such lack of mitochondrial dysfunction could be due to the type of tissue studied. Cryopreserved homogenates from colonic biopsies were used in the experimental procedures. In preliminary studies, only a few cells could be cultured and could have undergone cell death before reaching optimal conference. Other authors were successful in obtaining neuronal cultures from the enteric system by using the entire myenteric plexus, in human fetal tissue [37] or in mouse in vitro models [38, 39]. In the present study, the amount, type, or location of the biopsies (small, with heterogeneous cell types, and superficial) could have hampered either the cell growth in the culture of enteric neurons or the detection of significant mitochondrial disturbances between groups.

Our gastrointestinal biopsies contain epithelial cells, enteric glial cells, and enteric neurons. It is important to note that, aside from neurons, the enteric glial cells and epithelial cells, which are present in a gastrointestinal biopsy, are also dysregulated in PD [40]. We have proven the presence of enteric neuronal axons in our samples. According to the longitudinal location of the biopsies, despite less prevalence of Lewy pathology in the lower compared to the upper gut, the sigmoid colon and rectum have been by far the most commonly studied sites [41]. In this study, the mitochondrial parameters were assessed from the sigmoid section of the biologic material. According to these data, global mitochondrial findings herein depicted might be considered representative of expected alterations at PANS level in the context of PD.

Despite the negative results obtained, the trends observed towards suboptimal CI enzymatic activity in both groups of patients (iRBD and PD) point to the necessity of further research in mitochondrial studies in PANS. Such studies should address the role of mitochondrial disturbances as potential PD in vivo biomarkers in different sites and depths of enteric PD biopsies in larger cohorts of patients.

## Supplementary Material

The axonal presence in a colonic sample is shown in the image from confocal microscopy after immunohystochemistry (PGP.9.5 antibody).

## Figures and Tables

**Table 1:** Demographic and clinical data of the subjects included in the study.

	PD	iRBD	Control
Number of patients (M/F)	9 (7/2)	7 (6/1)	9 (4/5)
Age at biopsy (years, median, min-max)	65.18 (54.04–71.02)	72.44 (66.85–83.40)	64.79, (58.34–82.74)
Disease duration (years, median, min-max)	5.77 (0.76–13.81)	10.54 (1.64–22.25)	—
Hoehn and Yahr (median, min-max)	2 (1-2)	—	—
UPDRS I (median, min-max)	10 (1–19)	5 (1–17)	4 (0–9)
UPDRS II (median, min-max)	8 (2–14)	1 (0–8)	0 (0–3)
UPDRS III (median, min-max)	14 (5–34)	4 (1–27)	0 (0–4)
NMSQuest, gastrointestinal subdomain (median, min-max)	6 (0–16)	0 (0–9)	0.5 (0–12)
