# Supplementary material for: Colonic Oxidative and Mitochondrial Function in Parkinson's Disease and Idiopathic REM Sleep Behavior Disorder
Source: Parkinsons Dis. 2017 Jun 4;2017:9816095. doi: 10.1155/2017/9816095 (PMC5474269; doi:10.1155/2017/9816095)
Supplement: Supplementary file 1 — The axonal presence in a colonic sample is shown in the image from confocal microscopy after immunohystochemistry (PGP.9.5 antibody). [file 9816095.f1.pdf]

S1

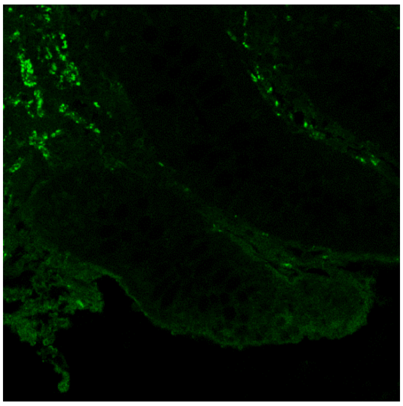

Supplementary Figure 1 S1. Micrography from colonic sample. Immunohystochemistry from a colonic sample using PGP.9.5 antibody against axonal neurons using confocal microscope.
